# *Plasmodium falciparum* Histidine-Rich Protein 2 and 3 Gene Deletions and Their Implications in Malaria Control

**DOI:** 10.3390/diseases8020015

**Published:** 2020-05-20

**Authors:** Josphat Nyataya, John Waitumbi, Victor A. Mobegi, Ayman Noreddin, Mohamed E. El Zowalaty

**Affiliations:** 1 Basic Science Laboratory, US Army Medical Research Directorate-Africa/Kenya Medical Research Institute, P.O. Box 54-40100, Kisumu, Kenya; Josphat.Nyataya@usamru-k.org; 2 Department of Biochemistry, School of Medicine, University of Nairobi, P.O. Box 30197-00100, Nairobi, Kenya; vatunga@uonbi.ac.ke; 3 Infectious Diseases and Anti-Infective Research Group, College of Pharmacy and Sharjah Medical Research Institute, University of Sharjah, Sharjah 27272, UAE; anoreddin@sharjah.ac.ae; 4 Department of Medicine, School of Medicine, University of California, Irvine, CA 92868, USA; 5 Zoonosis Science Center, Department of Medical Biochemistry and Microbiology, Uppsala University, SE-75123 Uppsala, Sweden

**Keywords:** malaria diagnosis, control, Africa, Kenya, *Plasmodium falciparum*, histidine-rich proteins, deletions, epidemiology

## Abstract

Malaria remains the biggest threat to public health, especially among pregnant women and young children in sub-Saharan Africa. Prompt and accurate diagnosis is critical for effective case management and detection of drug resistance. Conventionally, microscopy and rapid diagnostic tests (RDTs) are the tools of choice for malaria diagnosis. RDTs are simple to use and have been extensively used in the diagnosis of malaria among travelers to malaria-endemic regions, routine case management, and surveillance studies. Most RDTs target the histidine-rich protein (*Pf*HRP) which is exclusively found in *Plasmodium falciparum* and a metabolic enzyme *Plasmodium* lactate dehydrogenase (*p*LDH) which is common among all *Plasmodium* species. Other RDTs incorporate the enzyme aldolase that is produced by all *Plasmodium* species. Recently, studies have reported false-negative RDTs primarily due to the deletion of the histidine-rich protein (*pfhrp2* and *pfhrp3*) genes in field isolates of *P. falciparum*. Herein, we review published literature to establish *pfhrp2/pfhrp3* deletions, the extent of these deletions in different geographical regions, and the implication in malaria control. We searched for publications on *pfhrp2/pfhrp3* deletions and retrieved all publications that reported on this subject. Overall, 20 publications reported on *pfhrp2/pfhrp3* deletions, and most of these studies were done in Central and South America, with very few in Asia and Africa. The few studies in Africa that reported on the occurrence of *pfhrp2/pfhrp3* deletions rarely evaluated deletions on the flanking genes. More studies are required to evaluate the existence and extent of these gene deletions, whose presence may lead to delayed or missed treatment. This information will guide appropriate diagnostic approaches in the respective areas.

## 1. Introduction

The previous two decade was characterized by remarkable progress in the fight against malaria, with more than seven million lives having been saved since 2001 [1], thanks to the wide-scale deployment of malaria control interventions, including but not limited to accurate diagnosis, effective antimalarial therapy, and use of insecticide-treated nets [2]. However, recent reports suggest that these gains are slowing down, and this may reverse all the investments and efforts that have been utilized so far [3]. In 2015, an estimated 211 million cases of malaria were reported, and by 2018, the cases had increased to 228 million. More than 90% of these cases were reported in the African region. Globally, 405,000 deaths from malaria were reported in 2018, with 17 African countries accounting for 91% of all malaria deaths [4]. Children under five years of age and pregnant women remain the population at the highest risk of malaria.

Diagnosis of malaria requires the identification of *Plasmodium* parasites, which is usually done by the use of microscopy or rapid diagnostic tests (RDTs) that target antigens/enzymes of the parasite in the patient’s blood. Although molecular techniques such as Polymerase Chain Reaction (PCR) provide high sensitivity and specificity for malaria diagnosis, the high cost, limited availability, and required expertise make them unsuitable as a diagnostic tool, especially in resource-limited settings [5,6].

Recently, the use of non-invasive specimens such as saliva and urine has been proposed as an alternative to blood samples. However, the utility of these approaches has not been validated [7,8]. Microscopy and RDTs, therefore, form a core component of malaria diagnosis and case management. For effective case management, diagnosis of malaria has to be prompt and accurate. Accurate diagnosis ensures that antimalarial therapy is used rationally and correctly, malaria does not progress to complications such as severe anemia, metabolic acidosis, and cerebral involvement, and reduces the chance of transmission of the disease to other people. However, the performance of malaria RDTs is influenced by several factors—the existence of different species of *Plasmodium*, which requires expert microscopists to differentiate them, deletions of the antigens targeted by RDTs, transmission intensity, parasite density, immunity, and the manifestation of clinical symptoms that are similar to other tropical diseases [9,10]. Routinely, the diagnosis of malaria is based on both signs (clinical presentations/complains, fever measured by body temperature) and parasitological confirmation of parasites presence in blood [5].

Since Laveran discovered the malaria parasite and Romanowsky’s improvements on the staining techniques in the late 1800s, malaria has conventionally been diagnosed by the observation of *Plasmodium* parasites in slides stained with Giemsa or Field’s stain [11]. Microscopic examination of Giemsa-stained thick films (for detection of the presence of the parasites) and thin films (for species identification) is the gold standard approach for diagnosis [5]. Malaria microscopy has low direct costs, is simple, allows differentiation of parasite species and developmental stages (e.g., gametocytes), and allows determination of parasite density. These characteristics of microscopy make it an attractive tool for malaria diagnosis and drug efficacy studies [12]. However, despite its widespread use in malaria diagnosis, microscopy has several inherent limitations, including its dependence on the availability of a competent microscopists, electricity, good quality staining reagents, and enough parasite density (>100 parasites/µL) [13,14,15]. Microscopy is also labor-intensive, unsuitable for high-throughput use, and has a high error rate. The errors lead to biased estimates on measures of protective efficacy or misclassification of treatment outcomes, especially in antimalarial drug or vaccine trials. Studies comparing field-based and expert microscopy to PCR for detection of *Plasmodium* have shown that mixed species are common and are rarely detected by expert microscopists [16]. In rural settings with limited resources such as electricity and sources of good-quality water to make the stains, microscopy may not be reliable [17], and therefore, there is a need for other alternative diagnostic tools to complement microscopy.

To overcome the apparent limitations of microscopy, the World Health Organization (WHO) has emphasized the urgent need for new, simple, quick, accurate, and cost-effective diagnostic tools to complement malaria microscopy [6]. The advent of RDTs has greatly enhanced the quality of malaria diagnosis, especially in peripheral health centers where microscopy would not be reliable. Through global fund financing, over 148 million RDTs have been procured in 81 malaria-endemic countries, thereby improving case management, especially in children, who are the most vulnerable group [18]. The WHO Africa region, where the disease burden is highest, reported an increase in RDT sales up to 269 million from 240 million in 2015 [2]. RDTs are fast, easy to perform and interpret, do not need for electricity or equipment, and this has contributed to their widespread use.

Currently, there are about 60 manufacturers and 200 types of RDTs that have been tested and deployed in field settings [19]. All RDTs detect malaria antigen in blood sample placed on a lateral flow chromatographic membrane containing immobilized antimalaria antibodies. The dye-labeled antibody binds the target parasite antigen, and the resulting complex is subsequently captured by another antibody, which is immobilized at the test line. The color appears at the test line. Excess labeled antibody conjugate continues to flow along the strip and is captured at the control line by a secondary antibody. When a sample without target antigens is used, color appears only on the control line.

Malaria RDTs target three *Plasmodium* antigens that are detected in the same RDT. The histidine-rich proteins 2 and 3 (*Pf*HRP2/3) are specific for *P. falciparum*, while *Plasmodium* lactate dehydrogenase (*p*LDH) and *Plasmodium* aldolase are made by all malaria species, hence the name pan-*p*LDH and pan-aldolase. Thus, in general, RDTs are sold as combinations of pan-*p*LDH-*Pf*HRP2/3 or pan-aldolase-*Pf*HRP2/3 [5].

The *pLDH* is a terminal enzyme in the glycolytic pathway of both the sexual and asexual stages of the parasite, and each of the four *Plasmodium* species has its specific isomer of this enzyme, while aldolase is conserved in all the species and is an example of a pan-malarial antigen target [20]. *p*LDH catalyzes (using NADH, a reduced form of nicotinamide adenine dinucleotide, as a coenzyme) the conversion of pyruvate into lactate and NAD^+^, (oxidised form of NAD) which is important for parasite survival in red blood cells [21,22]. Since *p*LDH and aldolase are only produced in living *Plasmodium* cells and have a short half-life (2–4 days), the presence of these enzymes is an indication of current infection [23]. RDTs that detect *p*LDH either detect a pan-*p*LDH which is common to all human infecting species or specific regions unique to *P. falciparum* or *P. vivax*-specific *p*LDH [24]. Although previous studies showed reduced sensitivity of most *p*LDH-detecting RDTs at extreme temperatures [25], a study in India demonstrated RDT stability and high sensitivity of up to 98% for *P. falciparum* [26].

Three histidine-rich proteins (HRPs) have been described, namely, HRP1 or the knob-associated histidine-rich protein (KAHRP), HRP2, and HRP3, also known as small histidine–alanine-rich proteins (SHARP) containing 8%, 35%, and 30% histidine repeats, respectively [27,28]. The *pfhrp1* gene is associated with the expression of knob-like protrusions on the surface membrane of infected erythrocytes [29]. These knobs are not seen in erythrocytes infected with the ring stages of the parasites, but they develop with the maturation of the parasites to other developmental stages, such as late trophozoites and schizonts [30]. Studies have revealed that the knobs are the sites of attachment to the venous endothelium and are thus responsible for sequestration of infected erythrocytes to deep vasculature, a phenomenon that is common in cerebral malaria [31].

*Pf*HRP2 is a water-soluble protein produced by the asexual stages and young gametocytes of *P. falciparum* and can be detected in serum, plasma, cerebrospinal fluid, and urine of infected patients and in the medium of *in vitro* cultured parasites [32,33,34]. Secretion and synthesis of *Pf*HRP2 have been shown to begin with immature rings and increase gradually as the asexual parasites mature such that, more than 90% is secreted during schizogony [35,36], and its presence in plasma has been exploited in the design of RDTs for malaria diagnosis [37]. Unlike *Pf*HRP1, which is found only in knobby (K+) variants of *P. falciparum*, *Pf*HRP2 is found in both the knobby and knobless (K-) strain variants [38].

The *pfhrp3* gene codes for a homologous protein histidine-rich protein-3 (*Pf*HRP3). Both *Pf*HRP2 and *Pf*HRP3 are very similar in structure, although *Pf*HRP3 has less histidine content [27]. The structural similarity between the two genes is responsible for cross-reaction of monoclonal antibodies against *Pf*HRP2 with those of *Pf*HRP3. Unlike *Pf*HRP1 and *Pf*HRP3, *Pf*HRP2 is abundantly produced and is continuously expressed throughout the parasite life cycle.

**Figure 1:**
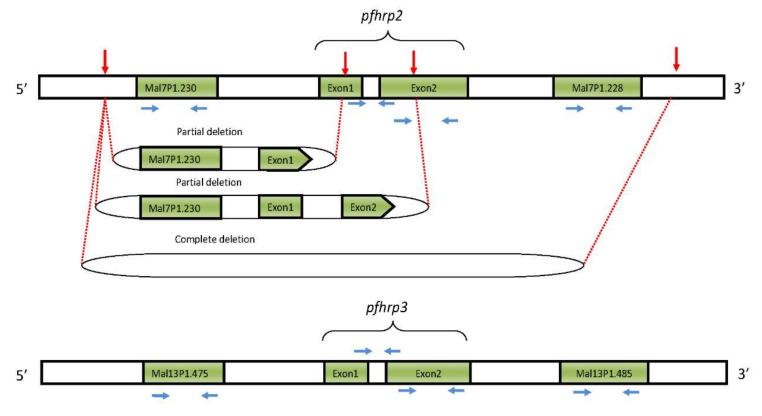
Illustration of *pfhrp2 and pfhrp3* genes and their flanking regions (green color), chromosome breakage and rejoining points (in red arrows) and the primer locations for the target genes (blue arrows).

The use of *Pf*HRP2-based RDTs for malaria diagnosis has emerging limitations due to the existence of parasites with *pfhrp2* gene deletions which, depending on the extent of the deletions, has led to false-negative results [39,40,41,42]. Initially, experimental studies demonstrated the deletions of *pfhrp2* and *pfhrp3* genes in laboratory lines [43,44]. Whole-genome scanning and DNA sequence analysis have demonstrated partial or complete deletions of *pfhrp2/pfhrp3* genes and/or the flanking genes [40,45]. The deletions involve breakage and rejoining at the unstable sub-telomeric regions of chromosome 8 and 13 for *pfhrp2* and *pfhrp3* genes, respectively (Figure 1).

Recently, studies have confirmed the existence of deletions in field isolates, thereby threatening the utility of *Pf*HRP2-based RDTs for malaria diagnosis. Depending on the extent of the deletions, the expression level of *Pf*HRP2 and *Pf*HRP3 proteins could cause false-negative results, leading to delay in therapy, and ultimately contribute to increased morbidity and mortality and promote disease transmission. Parasites with *pfhrp2* gene deletion have been shown to be reactive to *Pf*HRP2-based RDTs due to cross-reactivity with *Pf*HRP3 [46,47]. Few studies have evaluated the presence and extent of these deletions in Africa. In this review, we evaluate the current literature on *pfhrp2* and *pfhrp3* gene deletions and their potential impact on disease diagnosis, management, control and the eradication goals.

## 2. Methods

### 2.1. Systematic Review Protocols

The guidelines and procedures of the Preferred Reporting Items for Systematic Reviews and Meta-Analyses (PRISMA) [48] were followed in the current study (Figure 1). 

### 2.2. Search Strategy and Inclusion/Exclusion Criteria

Published scientific articles were retrieved from PUBMED, MEDLINE, and Science Direct. We searched the scientific literature for articles published in English from January 2010 when the first case of *P. falciparum* parasites lacking *pfhrp2* and/ or *pfhrp3* deletions was first reported [40], to December 2019.

Keywords used for the search included: *Pf*HRP2, *Pf*HRP3, *pfhrp2/pfhrp3* gene deletions, and *Pf*HRP2-RDTs. To filter out articles that were out of scope, we screened only for publications that were reporting on *pfhrp2/pfhrp3* deletions. To be included in the analysis plan, a study had to fulfill stipulations as previously suggested [49], i.e., have one initial evidence (microscopy or RDT or both) of the parasite and include a quality control procedure such as co-amplification of a single copy gene, in addition to amplification of *pfhrp2* and/or *pfhrp3* genes. This is crucial to allow differentiation between lack of amplification due to gene deletions versus no amplification due to PCR failure.

### 2.3. Statistical Analysis

Data were entered in a Microsoft Excel database (Microsoft, Redmond, WA, USA). The meta-analysis was conducted using the random effects model of analysis since it minimizes heterogeneity of the included studies. All of the analyses were implemented using R-3.6.2 metaprop and forest packages in order to estimate the pulled proportion, to investigate publication and other bias and to summarize information on individual studies, respectively. Descriptive statistics, such as bar charts, were used to summarize the distribution of reported deletions using the Statistical Package for Social Sciences (SPSS), version 25.

## 3. Results and Discussion

### 3.1. pfhrp2/pfhrp3 Deletions

**Figure 2:**
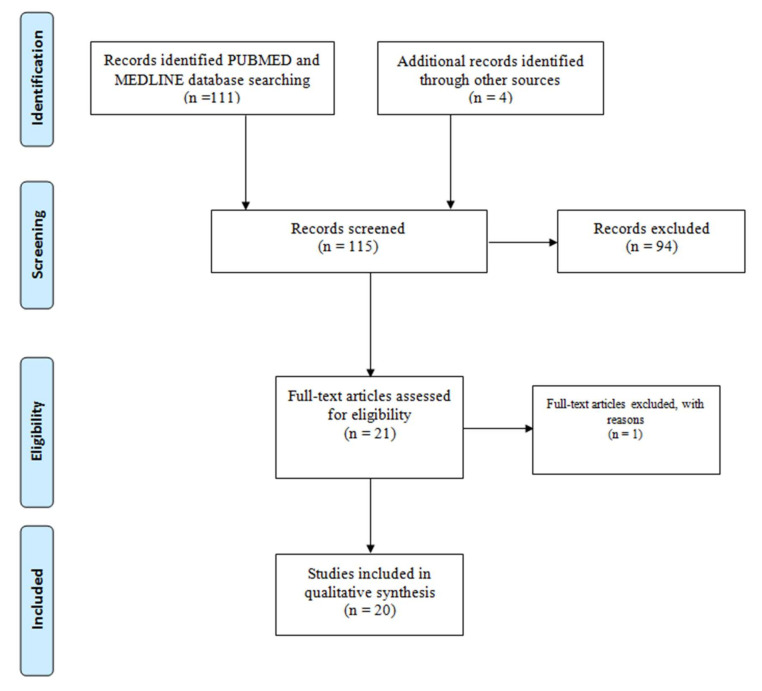
Preferred Reporting Items for Systematic Reviews and Meta-Analyses (PRISMA) flow chart showing the screening and retrieval process of publications that were eventually used in the analysis.

Figure 2 summarizes the screening and retrieval process for the publications reporting *pfhrp2* and *pfhrp3* deletions. The final number of 20 publications that were reviewed is summarized in Table 1.

Gamboa et al. (2010) reported the first evidence of field isolates lacking *pfhrp2* and *pfhrp3* genes in Peru and demonstrated that these parasites were widespread across the Peruvian Amazon [40]. A huge proportion of the retrospectively collected samples were found to lack the *pfhrp2* (41%), *pfhrp3* (70%) or both *(*21.6%) genes. The loss of these genes was confirmed by inability to detect the proteins in an immunological assay [40]. A similar study by Akinyi et al. (2013) in the same region of Peru demonstrated a 20% to 40% increase in the frequency of malaria parasites lacking *pfhrp2* over a seven year period [50].

*Pfhrp2* and *pfhrp3* gene deletions have also been reported in *P. falciparum* isolates from Colombia within the Amazon River region, like in Peru [51,52], where the prevalence of *pfhrp2* deletions was lower than that of *pfhrp3* (18% and 52%, respectively). This is not surprising considering that the majority of parasites with *pfhrp2* deletions were collected at the Peru border, where *pfhrp2* deletions had been reported before. A study that evaluated the population structure of Colombian *P. falciparum* at the Colombian pacific coast revealed four different subpopulations (Col-1, Col-2, Col-3, and Col-4) [67]. Similarly, the study by Solano et al. (2015) showed that the parasite isolates segregated into four clusters according to geography (Cluster 2 consisted 100% of the isolates from the Amazonas department, 59% of the isolates from Nariño department segregated into Cluster 4, 68% of isolates collected from Valle were assigned to Cluster 3, and 75% from Cordoba were assigned to Cluster 1). A total of 67% of the *pfhrp2* deleted parasites and 61.5% of the *pfhrp2*/ *pfhrp3* double negatives were found to segregate in Cluster 2, which consisted of samples mostly collected from the Amazonas department that borders Peru and Colombia within the Amazon ecological zone, indicating expansion and a common genetic origin of *pfhrp2* and *pfhrp3* deleted parasites [51].

Although earlier studies in Honduras only reported *pfhrp3* and not *pfhrp2* deletions, recent reports have documented extensive deletions of both genes [39,54]. The frequency of parasites lacking *pfhrp2* and *pfhrp3* in South and Central Americas has increased over time, and there is a need to evaluate the selective pressures that favor the survival of parasites that lack these genes [41].

**Figure 3:**
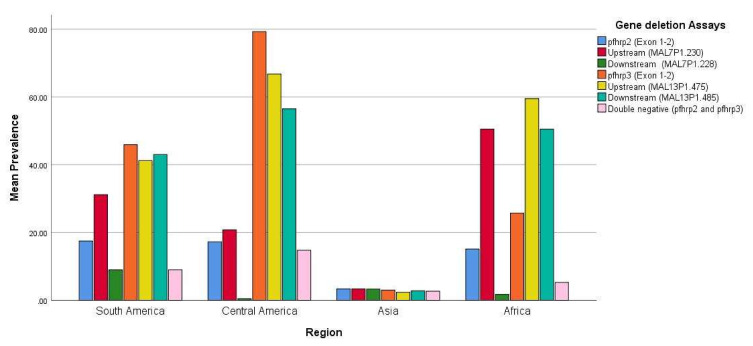
Bar chart showing the mean prevalence and distribution of *pfhrp2, pfhrp3*, and the corresponding flanking gene deletions in South America, Central America, Asia, and Africa.

Studies in India reported similar frequencies of parasites lacking *pfhrp2* and *pfhrp3* and their corresponding flanking regions [55,56,57]. Figure 3 summarizes the prevalence and distribution of *pfhrp2, pfhrp3* and the flanking gene deletions in different continents. As shown in Figure 3, the lowest prevalence of *pfhrp2/pfhrp3* and the respective flanking gene deletions was reported in Asia. In one of the studies, deletions of the two genes were observed in symptomatic individuals with high parasitemia [56], contrary to previous observations of *pfhrp2* and *pfhrp3* deletions in asymptomatic individuals with low parasitemia [60].

In Africa, where malaria is endemic and the use of RDTs is widespread, there is scarce information on *pfhrp2/3* deletions, although a few surveillance studies have reported on their existence [60,61,63,68,69]. 

The first cases of *pfhrp2* gene deleted parasites were reported in Mali, where deletions were identified in parasitemic individuals whose blood was unreactive to *Pf*HRP2-based RDTs [60]. In that study, the participants were asymptomatic and the parasites had low multiplicity of infection, a term used to describe strain diversity in an infected individual. Although parasite density may have influenced the variability observed in *Pf*HRP2-based RDTs, Wurtz et al. (2013) did not find any difference in parasitemia between groups with *pfhrp/pfhrp3* deletions and those without deletions, suggesting that, in addition to parasite density, lack of expression of the *Pf*HRP2 may greatly influence the performance of RDTs. A recent study in Eritrea reported the emergence of false-negative *Pf*HRP2-based RDTs that was not related to poor quality of RDTs, storage, handling or operator errors [70]. A subsequent study in the same population revealed that most patients were infected with parasites that were lacking *pfhrp2* (62%) and *pfhrp3* (82%), and that these parasites may have emerged due to local selective pressure since the parasite clusters obtained were not related to South American strains [66].

In Kenya, one study has reported the presence of *pfhrp2* deleted parasites in samples collected from asymptomatic children [12]. Curiously, these *pfhrp2* deleted samples were positive by *Pf*HRP2-based RDTs. Although this phenomenon was not reported in the South and Central America studies, it may be due to close reaction of *Pf*HRP2 monoclonal antibodies with *Pf*HRP3. *In vitro* studies have confirmed the detection of parasites with deleted *pfhrp2* on *Pf*HRP2-based RDTs, but this has not been reported widely in field samples [46]. Because of potential failures of RDTs, studies have recommended the performance of two RDTs in parallel as an initial screening test when evaluating *pfhrp2/pfhrp3* deletion [49].

**Figure 4:**
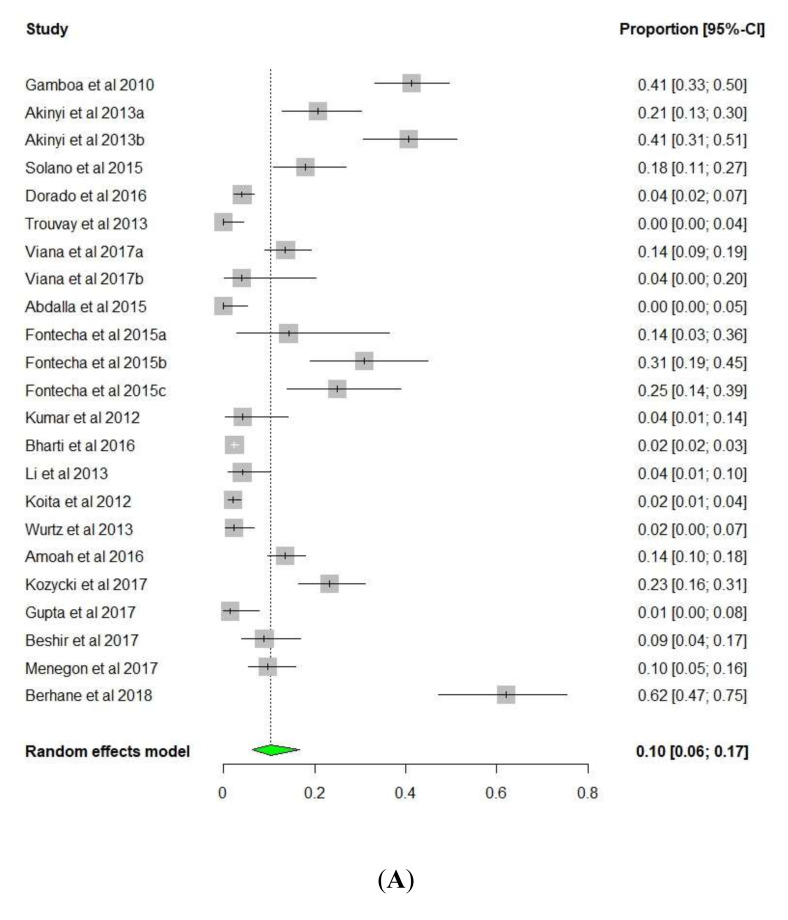

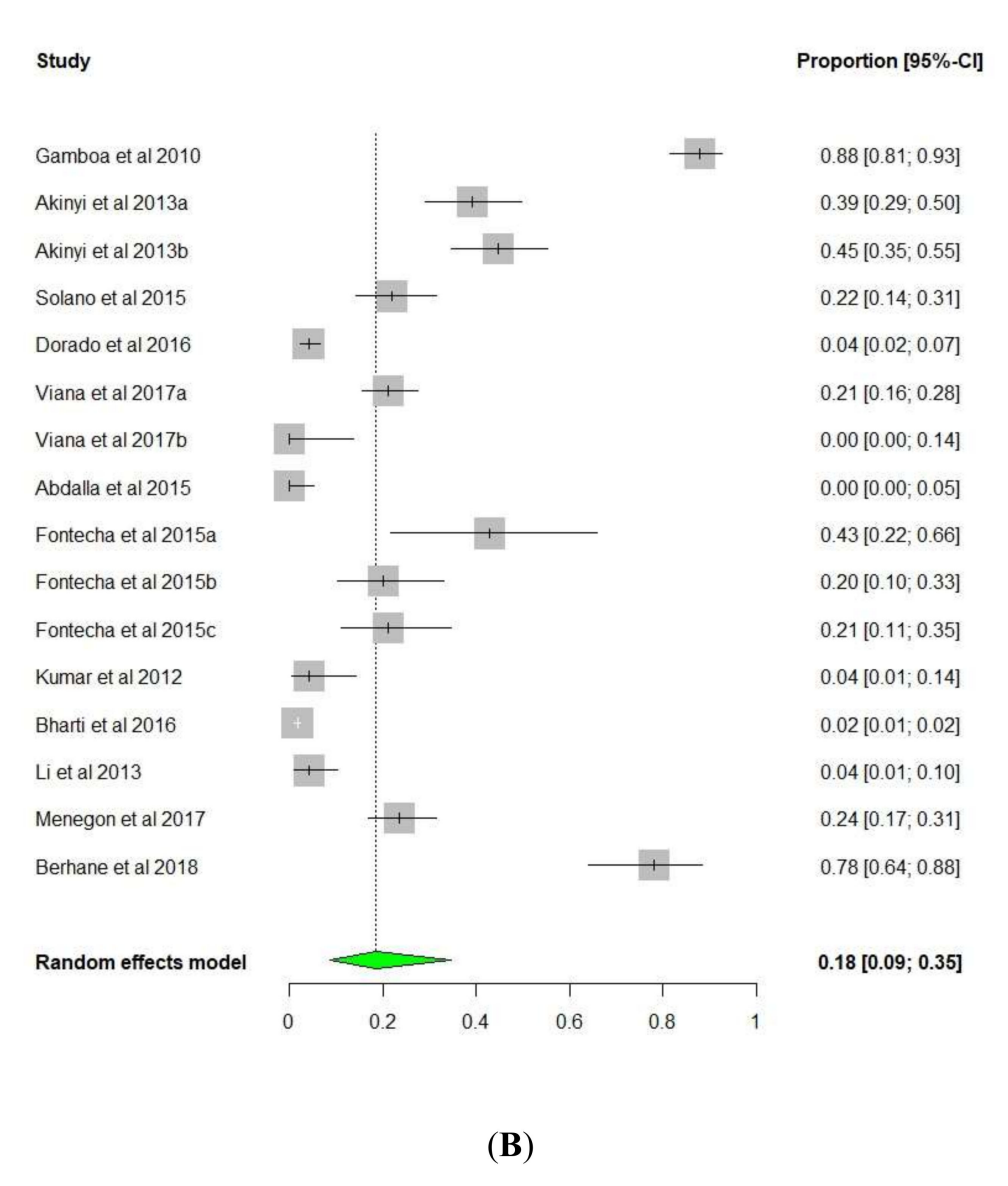

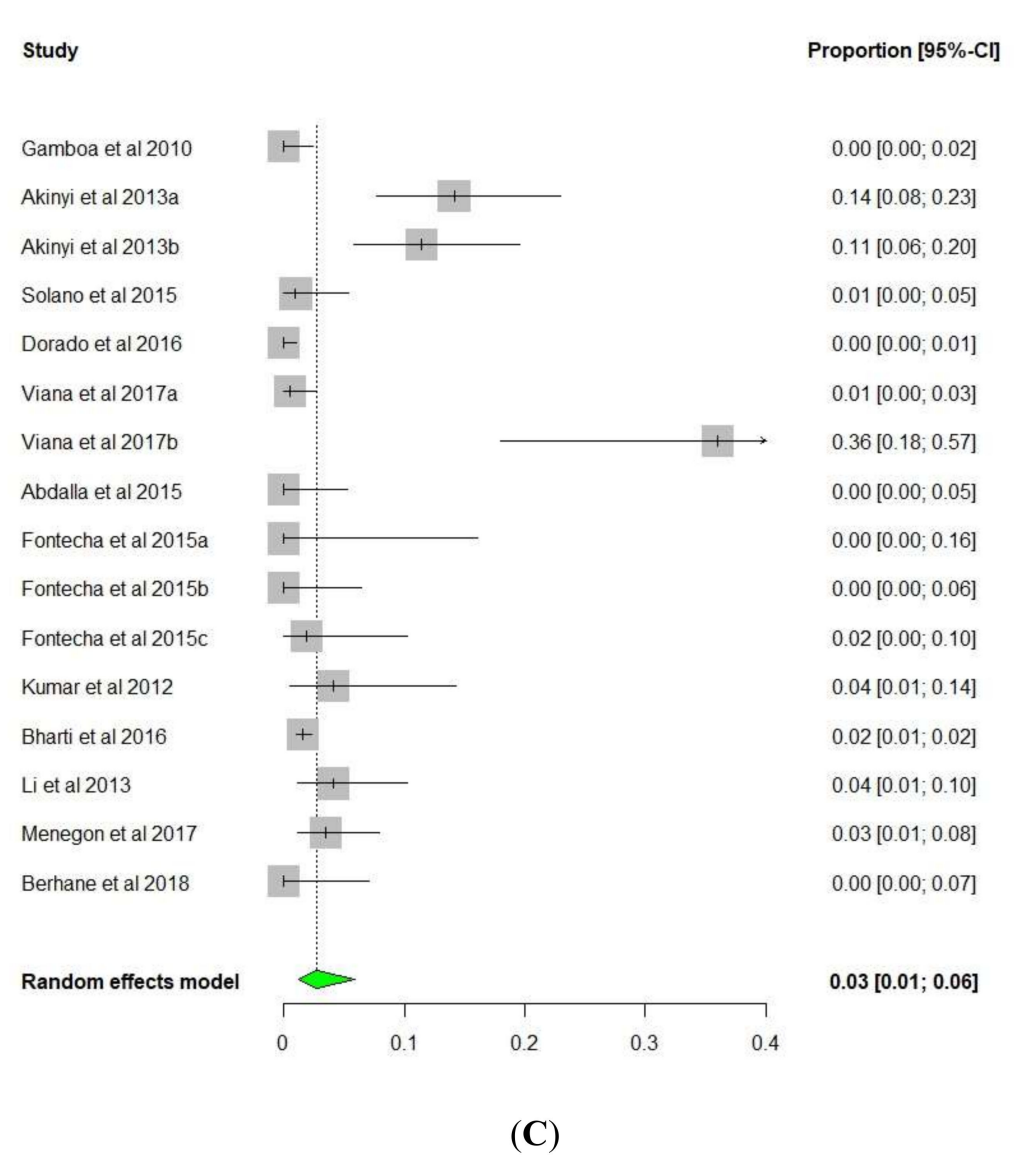

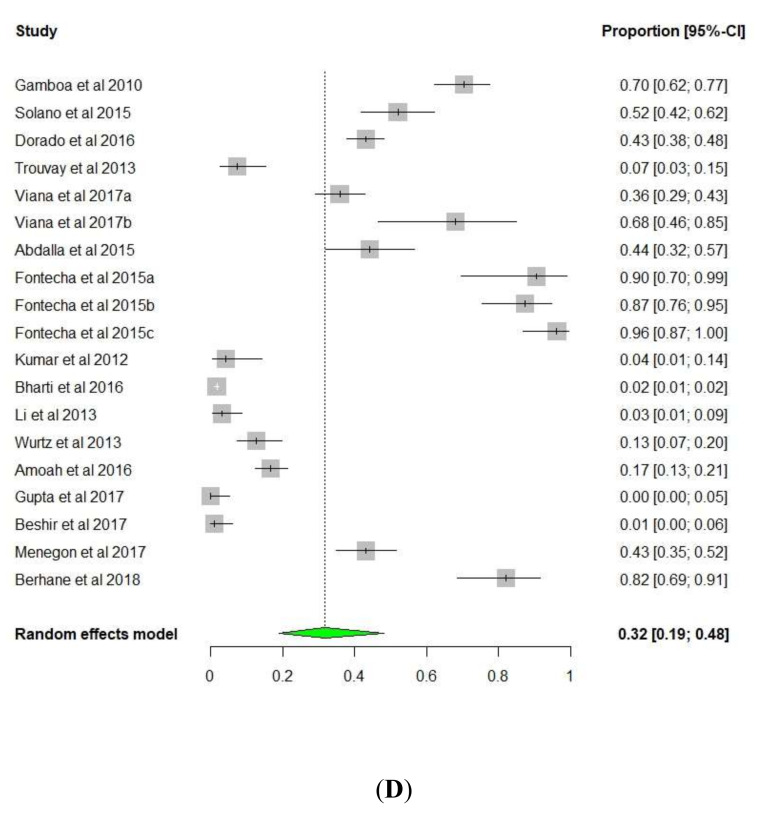

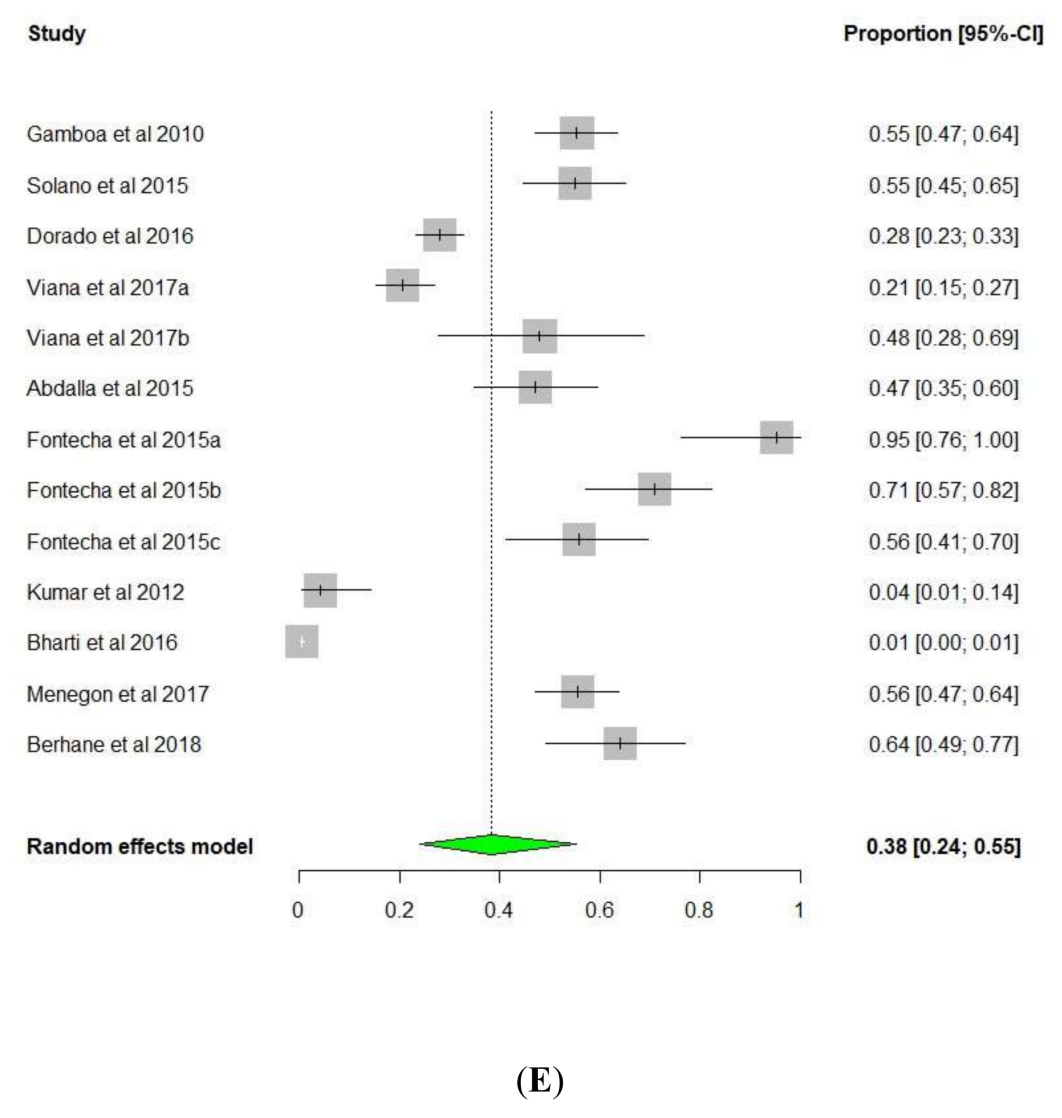

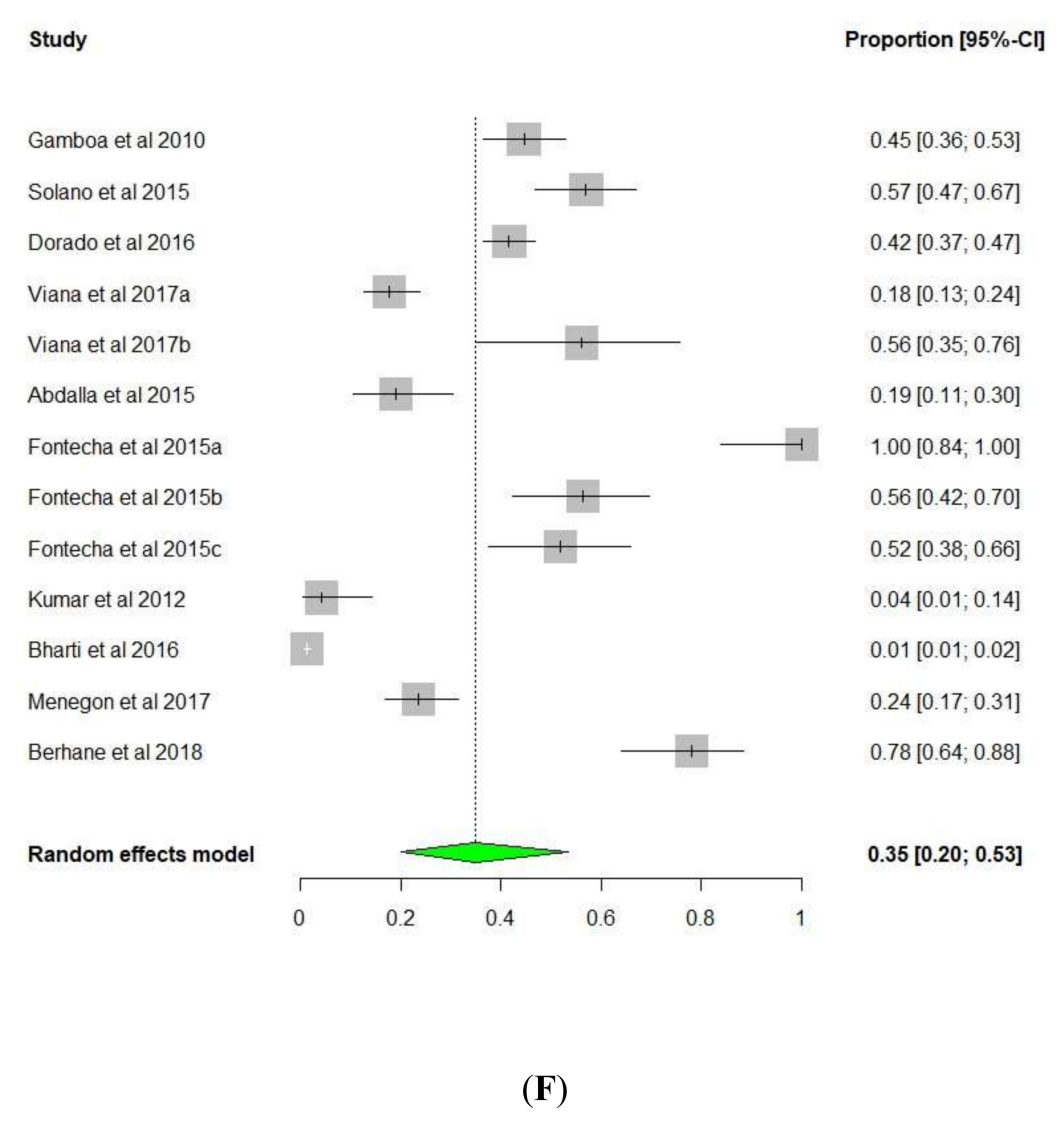

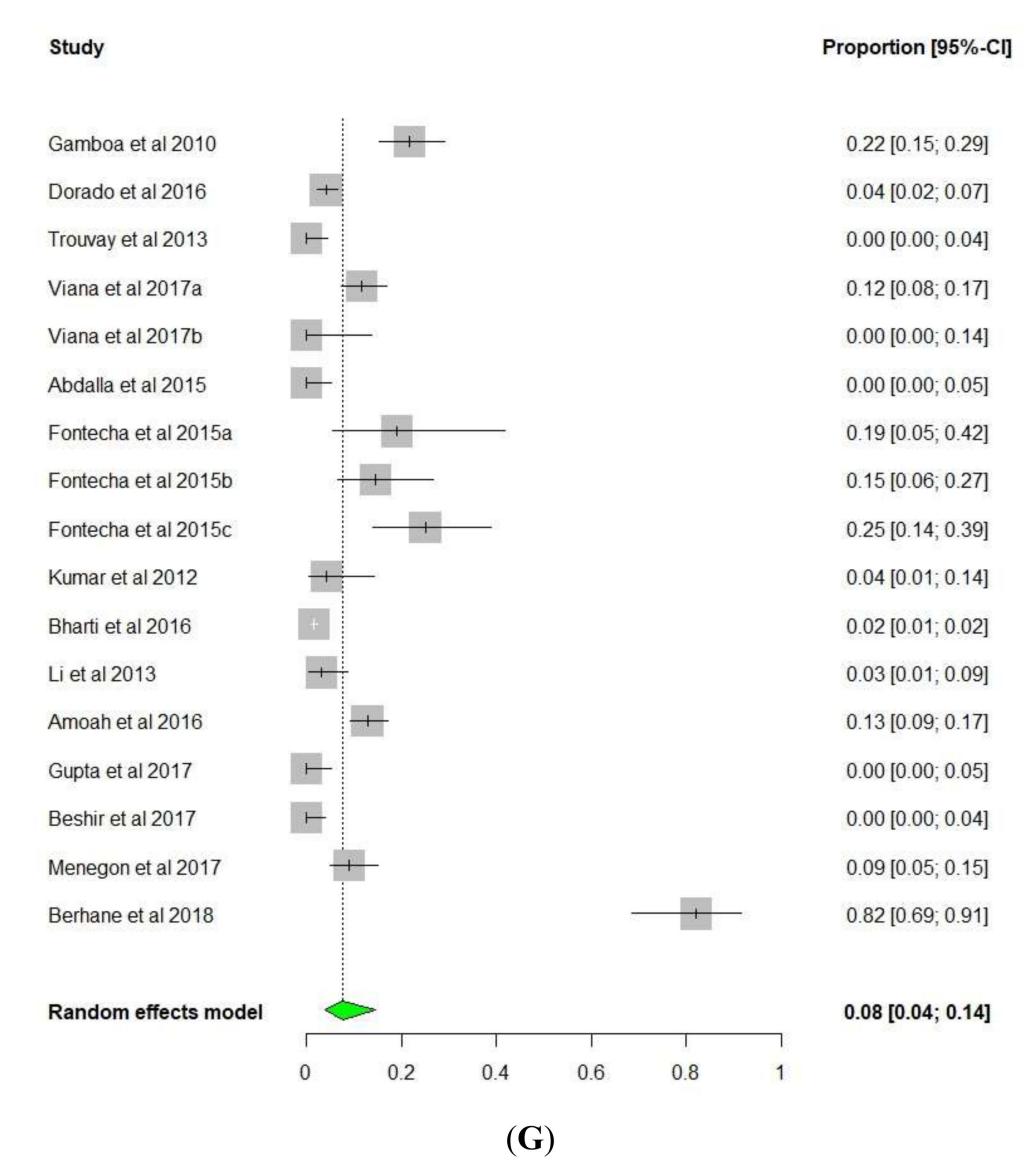
Forest plot showing the prevalence of (**A**): (*pfhrp2* exon1-2) (**B**): (Upstream *MALP1.230*) (**C**): (Downstream *MALP1.228*) (**D**): (*pfhrp3* exon1-2), (**E**): (Upstream *MAL13P1.475*) (**F**): (Downstream *MAL13P1.485*), and (**G**): (Double negative for *pfhrp2* and *pfhrp3*).

Overall, *pfhrp2/pfhrp3* deletions have been widely reported in Central and South America, where most studies reported a higher prevalence of *pfhrp3* (Lowest = 7.4% in French Guiana and Highest = 96.2% in Honduras) than of *pfhrp2* (Lowest = 4.0% in Bolivia and Highest = 40.6% in Peru). Similar deletions were also observed for the upstream and downstream regions flanking *pfhrp2* and *pfhrp3* genes (Table 1 and Figure 3). The studies included in the current analysis showed extensive heterogeneity in reporting the prevalence of *pfhrp2/pfhrp3* and the flanking genes (Figure 4). This could partly be attributed to the small sample size in these studies. We did not find any publication that reported a lack of deletion in all the genes of interest. 

The results of the meta-analysis with random effects model are presented in Figure 4. Based on the estimates, we noted that the proportion of *pfhrp2* exon1-2 among the included studies ranges from a minimum of 0.01 (95% CI: 0.00, 0.04) [53] to a maximum of 0.62 (95% CI: 0.47, 0.75) [66]. Furthermore, the pooled proportion of *pfhrp2* exon1-2 was 0.10 (95% CI; 0.06, 0.17) (Figure 4A). The heterogeneity test showed presence of lack of uniformity, *p*-value = < 0.001. Similarly, the pooled prevalence of Upstream MALP1.230, Downstream MALP1.228, *pfhrp3* exon1-2, Upstream MAL13P1.475, Downstream MAL13P1.485 and Double negative for *pfhrp2* and *pfhrp3* were 18.00%, 3.00%, 32.00%, 38.00%, 35.00% and 8.00%, respectively (Figure 4B-G).

Cheng et al.(2014) published a set of recommendations for reporting *pfhrp2/pfhrp3* deletions [49]. These recommendations include an initial screening whereby the sample is confirmed positive for *P. falciparum* by two expert microscopists and PCR. This is followed by a confirmatory evidence of deletion through PCR amplification of exon 2 of *pfhrp2* and *pfhrp3* and the region across exon 1 and 2 of both genes.

For quality control, it is also recommended to include PCR amplification of single copy genes such as *msp1* and *msp2* to rule out negative results due to low-quality DNA or PCR failure. We used these recommendations to evaluate which studies conformed to this protocol. All studies conducted in South America, Central America, and Asia conformed to these recommendations for reporting *pfhrp2/pfhrp3* deletions including PCR analysis of the flanking genes, performing RDTs on the suspected samples, and functional analysis through *Pf*HRP2 ELISA. These recommendations are also in agreement with the recently published WHO guidelines on reporting *pfhrp2/pfhrp3* deletions [71] and as such, should be adhered to for the validity of any study on *pfhrp2/pfhrp3* gene deletions.

Unfortunately, of the eight studies conducted in Africa, only two followed these guidelines. Most reported on the prevalence of *pfhrp2* (Lowest = 1.5% in Mozambique and Highest = 62.0% in Eritrea) and *pfhrp3* (Lowest = 1.1% in Kenya and Highest = 82.0% in Eritrea [12,62,63,64]. The two studies that included analysis of flanking genes in the detection of *pfhrp2/pfhrp3* deletion were from Eritrea. However, the study by Menegon et al. (2017) did not include RDT results [65].

Although the upstream and downstream flanking genes for *pfhrp2* and *pfhrp3* are not well characterized, they have been observed [49], and as such, it is recommended to include these genes when evaluating *pfhrp2* and *pfhrp3* gene deletions. As shown in Table 1, most studies did not conform to the WHO recommendation for accurate reporting of *pfhrp2/pfhrp3* deletions. This may affect the validity of the claims made [49,71].

Apart from gene deletions, sequencing of the *pfhrp2* and *pf*hrp3 genes has revealed extensive variability and different haplotype profiles in parasites from different geographical areas, and this may influence the performance of RDTs [72,73]. For example, the sequencing of these genes has revealed an extensive variation in the number of histidine repeats, copy numbers, and single nucleotide polymorphisms [58,74,75]. The functional utility of these variations needs further investigations, since this variability has not been shown to affect the amount of *Pf*HRP2 in plasma, which is the basis for parasite detection on RDTs [74,76].

### 3.2. Implication of pfhrp2/pfhrp3 Deletions on RDT Use

The extensive decline in malaria witnessed over the last two decades is attributed to improved diagnosis, effective treatment, and monitoring. The details of the decline are captured in the WHO Global Initiative for malaria control referred to as T3 (Test, Treat, and Track) which provided a framework for malaria control and elimination in malaria-endemic countries [77]. This initiative has contributed to the increased use of RDTs for diagnostic testing, especially in areas where microscopy is not available. 

Recent evidence of false-negative RDTs due to the emergence of parasites lacking *pfhrp2* and/or *pfhrp3* may negatively impact the success of RDTs in malaria diagnosis and could present a major threat to the progress made in the fight against malaria [40,49], and may delay eventual malaria eradication goals. Although the selective pressure-favoring survival of *pfhrp2* depleted parasites is not clearly defined, mathematical modeling has suggested that the use of *Pf*HRP2-based RDTs over the last decade favored the survival of parasites lacking *pfhrp2* genes. The model also suggests that selection pressure is high in areas where the prevalence of malaria is low, and the treatment of cases is based on RDT diagnosis [78]. This is because, in low transmission settings, most infections are asymptomatic and are characterized by low-density parasitemia, which is below the detection limit of the RDTs. This means that these infections are not treated and thereby enhance parasite survival [79,80]. Because of the structural homology of *Pf*HRP2 and *Pf*HRP3 antigens and the observed cross-reaction of monoclonal antibodies used in the RDTs, it would be expected that *Pf*HRP3 would compensate for the deletion of *pfhrp2*. However, because *Pf*HRP3 is not abundantly produced, this is likely to be beneficial only at high parasitemia [46]. 

The decline in malaria transmission that has been witnessed in most areas means that regions previously considered endemic may not experience as high a number of malaria cases as before, and most infections will likely be characterized by low-density parasitemia. If the model proposed by Watson is correct, this will increase the number of missed diagnosis by both microscopy and RDT; therefore, individuals harboring *pfhrp2* depleted parasites are likely to go undetected [78]. Consequently, this will likely amplify the population of malaria parasites with the these deletions [49]. In peripheral health care facilities where microscopy is not available or among travelers to malaria-endemic areas, a common practice is to rule out malaria in patients that test negative with an RDT. This practice could also aid in the selection of *pfhrp2* deleted malaria parasites. 

This review demonstrates the emerging threat of *pfhrp2/pfhrp3* deleted parasites mostly in areas that have had a history of *P. falciparum* malaria. This could become a big challenge, especially with the increased use of *Pf*HRP2-based RDTs. In sub-Saharan Africa, where malaria is still endemic, the extent of *pfhrp2/pfhrp3* gene deletions is underreported [71,81]. There is, therefore, a need for more extensive studies to evaluate the true prevalence of *pfhrp2/pfhrp3* deleted parasites and the impact they have on malaria diagnosis.

## Figures and Tables

**Table 1:** Summary of studies reporting *pfhrp2/hrp3* deletions.

Region	Country (Year of Sample Collection)	Initial Evidence		Gene Deletion Assays							
		RDT	Microscopy	*pfhrp2-*(Exon 1–2)	Upstream *MAL7P1.230*	Downstream *MAL7P1.228*	*pfhrp3-*(Exon 1–2)	Upstream *MAL13P1.475*	Downstream *MAL13P1.485*	Double Negative (*pfhrp2* and *pfhrp3*)	Reference
South America	Peru (2003–2007)	D	D	41% (61/148)	88% (8/9)	0	70% (103/148)	55.6% (5/9)	44.4% (4/9)	21.6% (32/148)	[40]
	Peru (1998–2001)	ND	ND	20.7% (19/92)	39.1% (36/92)	14.1% (13/92)	ND	ND	ND	ND	[50]
	Peru (2003–2005)	ND	ND	40.6(36/96)	44.8(43/96)	11.5% (11/96)	ND	ND	ND	ND	
	Colombia (1999–2009)	D	D	18% (18/100)	22% (22/100)	1% (1/100)	52% (52/100)	55% (55/100)	57% (57/100)	ND	[51]
	Colombia (2003–2012)	D	D	4.1% (15/365)	4.1% (15/365)	0	43.0% (157/365)	28.0% (102/365)	41.6% (152/365)	4.1% (15/365)	[52]
	French Guiana (2010–2011)	D	D	0	ND	ND	7.4% (6/81)	ND	ND	0	[53]
	Brazil (2010–2012)	D	D	13.6% (27/198)	21.2% (42/198)	0.5% (1/198)	35.9% (71/198)	20.7% (41/198)	17.7% (35/198)	11.6% (23/198)	[42]
	Bolivia (2010)	D	D	4.0% (1/25)	0	36.0% (9/25)	68.0% (17/25)	48.0% (12/25)	56.0% (14/25)	NS	
Central America	Honduras (2008–2009)	ND	D	0	0	0	44.1% (30/68)	47.1% (32/68)	19.1% (13/68)	0	[39]
	Guatemala, 2015	ND	D	14.3% (3/21)	42.8% (9/21)	0	90.5% (19/21)	95.2% (20/21)	100% (21/21)	20% (11/55)	[54]
	Nicaragua, 2015	ND	D	30.9% (17/55)	20.0% (11/55)	0	87.3% (48/55)	70.9% (39/55)	56.4% (31/55)	14.3% (3/21)	
	Honduras (2011–2017)	ND	D	25.0% (13/52)	21.1% (11/52)	1.9% (1/52)	96.2% (50/52)	55.8% (29/52)	51.9% (27/52)	25% (13/52)	
Asia	India 2010	D	D	4.2% (2/48)	4.2% (2/48)	4.2% (2/48)	4.2% (2/48)	4.2% (2/48)	4.2% (2/48)	4.2% (2/48)	[55]
	India 2014	D	D	2.4% (36/1571)	1.7% (28/1571)	1.6% (26/1571)	1.7% (27/1571)	0.6% (10/1571)	1.4% (22/1571)	1.6% (25/1571)	[56]
	India (2018)	D	D	3.6% (38/1058)	0.5% (5/1058)	0.4% (4/1058)	2.3% (24/1058)	0.1% (1/1058)	0.3% (3/1058)	1.6% (17/1058)	[57]
	China–Myanmar (2011–2012)	D	D	4.1% (4/97)	4.1% (4/97)	4.1% (4/97)	3.1% (3/97)	ND	ND	3.1% (3/97)	[58]
	Bangladesh 2017	D	NS	CS	CS	CS	CS	CS	CS	CS	[59]
Africa	Mali (1996)	D	D	2.1% (10/480)	ND	ND	ND	ND	ND	ND	[60]
	Senegal (2009–2012)	D	D	2.4% (3/125)	ND	ND	12.8% (16/125)	ND	ND	NS	[61]
	Ghana (2015)	D	D	13.5% (29/288)	C	ND	16.7% (48/288)	ND	ND	12.9% (37/288)	[62]
	Rwanda (2014–2015)	D	D	23.1% (32/138)	ND	ND	ND	ND	ND	ND	[63]
	Mozambique (2010–2016)	D	D	1.5% (1/69)	ND	ND	0	ND	ND	0	[64]
	Kenya	D	D	9% (8/89)	ND	ND	1.1% (1/89)	ND	ND	0	[12]
	Eritrea (2013–2014)	ND	D	9.7% (914/144)	23.6% (34/144)	3.5% (5/144)	43% (62/144)	55.6% (80/155)	23.6% (34/144)	9% (13/144)	[65]
	Eritrea (2016)	D	D	62% (31/50)	78% (39/50)	0	82% (41/50)	64% (32/50)	78% (39/50)	82% (41/50)	[66]
